# Supplementary material for: Prevalence and factors associated with multidrug resistant Escherichia coli carriage on chicken farms in west Nile region in Uganda: A cross-sectional survey
Source: PLOS Glob Public Health. 2025 Jan 16;5(1):e0003802. doi: 10.1371/journal.pgph.0003802 (PMC11737776; doi:10.1371/journal.pgph.0003802)
Supplement: S1 File — (DOCX) [file pgph.0003802.s001.docx]

# **CONSENT FORM**

**Prevalence and factors associated with Multidrug Resistant *Escherichia coli* carriage on chicken farms in west Nile region in Uganda: A cross-sectional survey.**

Greetings to you. My name is Dr Ceaser Adibaku Nyolimati and I am a postgraduate student of the School of Public Health, in the College of Health Sciences, Makerere University, Kampala. My team is conducting a study aimed to determine *Escherichia coli* prevalence, antimicrobial resistance patterns and risk factors in Chicken farms in Arua district. This information will help the government to plan better healthcare services for the people of Uganda. The exercise on the farm may take around 45 minutes of your time. The team will ask questions on poultry management practices and would collect fresh faecal droppings on the farm from the farm-unit with the oldest flock of chickens. The samples will be tested for presence of *E. coli* organisms from the laboratory in Entebbe (National Animal Disease Diagnostic and Epidemiology Center). These bacteria are sometimes in chicken we keep without causing disease however, it can sometimes cause disease in chicken and people who are exposed to it. After the laboratory results are out, you will be told the result of this test. Sample collection may interrupt your chicken’s daily production cycle especially the layers. This study will take place between May and June 2023. We would very much appreciate your participation in this study. Whatever information you provide will be kept strictly confidential. Participation in this study is voluntary, and if we should come to any question, you do not want to answer, just let us know and we will go on to the next question. However, we hope that you will participate in this study since your views are important. For further information, please contact me (Dr Ceaser Adibaku Nyolimati) on Tel No: +256 774047958 or Email: cnyolimat@gmail.com

Would you like to participate in this study? Yes No

Signature of respondent: ______________________ Date: _______________

# **INTERVIEWER-ADMINISTERED QUESTIONNAIRE**

**Introduction**

The purpose of this questionnaire is to collect information that may assist in elucidating the risk factors that influences antimicrobial resistance in chicken and risk of transmission to humans.

**Investigator’s Details**

| Name of the investigator: |  |
| --- | --- |
| Contact of the investigator: |  |

**1. General farm characteristics**

| 1.1. Date of the survey: | 1.2. Time of visit: |
| --- | --- |
| 1.3. Farm identification code |  |
| 1.4. Location (GPS):  Lat:  Long: | 1.5. District:  1.6. Sub county:  1.7. Village: |
| 1.8. Name of the farmer: | 1.9. Contact of the respondent:  Tel No: |
| 1.10. Gender of the respondent: | 1.11. Role of the respondent on the farm: |

**2. Socioeconomic Characteristics of the Farmer/Farm Manager**

| 2.1 | Age |  |
| --- | --- | --- |
| 2.2 | Gender | 0= Female 1=Male |
| 2.3 | Marital status | 0=Single 1= Married |
| 2.4 | Education | 0=Informal/Adult Education  1=Primary Secondary  2=Tertiary |
| 2.5 | Primary profession | 0=Poultry farmer  1=Civil servant  2=Poultry seller  3=Poultry processor (Private sector)  4=Others Please specify ……………………… |
| 2.6 | How long have you been a poultry farmer? | 0=Less than 1 year 1=Up to 5 years  2=Up to 10 years 3=More than 10 year |
| 2.7 | What is the source of market for your chicken | 0=Export market  1=Domestic market |

**3. Management system**

| 3.1 | Chicken type | 0= Meat type (Broiler) 1= Egg type (Layer)  2=Dual purpose (Cross) |
| --- | --- | --- |
| 3.2 | Breed of the chicken kept | 0=Native 1=Exotics 2= Cross |
| 3.3 | Age category of the chicken | 0=Young 1=Adult  2=Market age 3=Unknown |
| 3.4 | Source of the chicken for the farm | 0=On farm  1=Feed and chick seller  2=Other poultry farmers |
| 3.5 | Production system | 0=Free range  1=Semi intensive  2=Intensive |
| 3.6 | Flock size | 0=Small holder farms (>50 – 100)  1=Medium size (101 – 1,000)  2=Large size (>1,001) |

**4. Biosecurity measures**

| 4.1 | How often do you clean the poultry litter? | 1=Every day 2=Twice a week  3=Once a week 4=Monthly |
| --- | --- | --- |
| 4.2 | How do you dispose the poultry manure? | 0=In pit  1=Use as fertilizer  2=Bag and sell |
| 4.3 | Do you wash your hands with soap after contact with poultry and their faeces? | 0=No  1=Yes |
| 4.4 | How do you dispose poultry by-products and other poultry wastes | 0=In pit  1=Use as fertilizer  2=Bag and sell |
| 4.5 | How frequent do you dispose poultry wastes on the farm | 1=Every day 2=Twice a week  3=Once a week 4=Monthly |
| 4.6 | Are there footbaths provided at entrance of the farm | 0=No  1=Yes |
| 4.7 | How frequent are disinfectants used in the footbath? | 0=Weekly  1=Biweekly  2=More than 2 weeks |
| 4.8 | Do the doors, windows and other openings of the houses have wire mesh? | 0=No  1=Yes |
| 4.9 | Is there a changing room on the farm/market | 0=No  1=Yes |
| 4.10 | Is there a lavatory on the farm? | 0=No  1=Yes |
| 4.11 | Do staff of the farm work on other poultry farms | 0=No  1=Yes |
| 4.12 | Are there other livestock species on the farm? | 0=No  1=Yes |
| 4.13 | Do you vaccinate your chickens | 0=No  1=Yes |
| 4.14 | What do you do with the chicken, which die on the farm? | 0=Dispose in a pit  1=Eaten by the family  2=Feed to other animals |
| 4.15 | Is the farm fenced? | 0=No  1=Yes |
| 4.16 | What is your source of drinking water? | 0=Tap water  1=Borehole  2=Well  3=Rivers and streams |

**5. Veterinary healthcare system**

| 5.1 | If your chickens are sick, who treats them? | 0=Government Veterinary doctor  1=Private Veterinary doctor  2= Feed chick dealer  3= Fellow farmer |
| --- | --- | --- |
| 5.2 | How often do you call them? | 0=Whenever needed  1=In case of ill health of the chicken |
| 5.3 | Are they easily accessible | 0=No  1=Yes |
| 5.4 | Do they provide the services you request? | 0=No  1=Yes |
| 5.5 | Do you know where their offices are? | 0=No  1=Yes |
| 5.6 | Do you have a nearby veterinary laboratory? | 0=No  1=Yes |
| 5.7 | If yes, is it functional? | 0=No  1=Yes |
| 5.8 | Are you charged for the services provided by the animal health workers? | 0=No  1=Yes |
| 5.9 | Are the numbers of qualified animal health workers enough according to your judgement? | 0=No  1=Yes |
| 5.10 | Who do you frequently consult in case your chicken are sick | 0=Government Veterinary doctor  1=Private Veterinary doctor  2= Feed chick dealer  3= Fellow farmer |

**6. Antimicrobial use**

| 6.1 | Can you show the drugs you used in the sampled shed in the current production cycle? |  |
| --- | --- | --- |
| 6.2 | What is the active ingredient of the drug? *(will be answered by the interviewer after they have a look at the drug)* |  |
| 6.3 | Picture of the drugs used *(if any)* |  |
| 6.4 | What is the purpose of using drugs in current batch of the poultry? | 0=Therapeutic 1=Prophylactic  2=Growth promotion 3=Both therapeutic  and preventive |
| 6.5 | How many times you use drugs in that sampled shed? | 0=Once daily 1=Twice daily  2=thrice daily |
| 6.6 | Is the dose same at each time? (this question is linked to question above) | 0=No  1=Yes |
| 6.7 | What is the route of administration? | 0=Feed 1=Water  2=Both feed and water |
| 6.8 | Do you stop using the drug before sale of birds? | 0= No  1= Yes |
| 6.9 | Who provided you with the drug? | 0=Pharmacy 1=Feed and chick dealer 2=Government Veterinarian |

**7. Monitoring and regulation**

| 7.1 | Do you get advices when using drugs on the farm? | 0=No  1=Yes |
| --- | --- | --- |
| 7.2 | Who advices you when using drugs | 0=Government Veterinary doctor  1=Private Veterinary doctor  2= Feed chick dealer  3= Fellow farmer |
| 7.3 | Do they come for follow-up after the advice? | 0=No  1=Yes |
| 7.4 | If yes, how often do they come? | 0=Daily  1=After another call |
| 7.5 | Do you consult qualified animal health worker before selling off your chicken? | 0=No  1=Yes |
| 7.6 | Do you know of any regulatory body for drugs? | 0=No  1=Yes |
| 7.7 | If yes, mentioned them. |  |

**8. Food supply system**

| 8.1 | Do you sell your chicken live? | 0=No  1=Yes |
| --- | --- | --- |
| 8.2 | If no, do you process it before sell? | 0=No  1=Yes |
| 8.3 | Do you have other workers responsible for sell of the chicken? | 0=No  1=Yes |
| 8.4 | Who are your main buyers of your chicken? | 0=Local community for domestic use  1=Restaurant operators  2=Supermarkets  3=Abattoir operators |
| 8.5 | Do you have other poultry farmers you sell your chicken? | 0=No  1=Yes |

**9. Farmers’ knowledge on AMR**

| 9.1 | Did you experience treatment failure on your farm? | 0=No  1=Yes |
| --- | --- | --- |
| 9.2 | What did you do when you experienced the treatment failure? | 0=No action taken  1=Consult veterinarian  2=Change drug used without consultation |
| 9.3 | Do you know that sometimes treatment can fail? | 0=No  1=Yes |
| 9.4 | Did you have any training on causes of treatment failure? | 0=No  1=Yes |
| 9.5 | How long have you been a poultry farmer? | 0=First time 1=less than 2 years  2=2 -5 years 3=More than 5 years |

**10. Withdraw period**

| 10.1 | How frequent do you treat your chicken? | 0=When they are sick,  1=When there is disease outbreak in the area,  2=When there is reduced production |
| --- | --- | --- |
| 10.2 | What did you do with chicken that died in the course of treatment? | 0=Dispose off  1=Eat it |
| 10.3 | What do you do in case you have a market for your chicken when they are on treatment? | 0=Do not sell  1=Sell it |

Thank you for your time.
