# Supplementary material for: Prevalence and factors associated with multidrug resistant Escherichia coli carriage on chicken farms in west Nile region in Uganda: A cross-sectional survey
Source: PLOS Glob Public Health. 2025 Jan 16;5(1):e0003802. doi: 10.1371/journal.pgph.0003802 (PMC11737776; doi:10.1371/journal.pgph.0003802)
Supplement: S3 File — (DOCX) [file pgph.0003802.s003.docx]

| BIOSAFETY MEASURES DURING SAMPLE COLLECTIONStandard Operating Procedure  \| SOP No. \| Document No /Version \| \| \| \| --- \| --- \| --- \| --- \| \| Title: \| Biosecurity and Biosafety practices \| \| \| \| Replaces Document: \|  \| Date of Revision \|  \| \| Applied to: Veterinary Microbiology Laboratories \| Reviewed & approved by: \|  \|  \| \| Date of Original \|  \| Date for next review \|  \|  1. **Introduction**   Good biosecurity practices must be employed when collecting samples from farms to avoid spreading disease from one farm to another. This should also be helpful in avoiding the bias that arises when farmers associate a disease outbreak with a sampling exercise that could have been carried out prior to that outbreak. Exposures to biological samples may cause serious infection, but effective treatment and preventive measures are available and the risk of spread of infection is limited.  It is therefore important for the persons collecting samples to be aware that he/she can potentially get infected when collecting samples from farms or from the environment as well as infect the farm or environment in which he/she collects samples. Appropriate biosafety and biosecurity measures should therefore be followed when collecting samples.   1. **Purpose**   The purpose of this Standard Operating Procedure (SOP) is to describe the procedures for best biosafety and biosecurity practices during sample collection on poultry farms targeted to protect the sampler, chicken and the farm. These samples should be used for isolation of *Escherichia coli*. The isolates should be subjected to antimicrobial susceptibility testing against the commonly used antimicrobials in line with the Clinical and Laboratory Standards Institute (CLSI) recommendations.   1. **Responsibilities**   The sampling team shall comprise of experienced veterinarians and laboratory technologists. The team shall ensure that strict biosecurity and biosafety measures are ensured following the SOP.   1. **Materials and equipment**  \| 1. Personal Protective Equipment (PPE). The minimum recommended PPE should include: hand gloves, gumboots, overall, disposable plastic apron, head net and masks 2. Sterile swab containers with Amies transport media containing charcoal 3. Cooler box(es) 4. Frozen ice packs \| 1. Paper towels 2. Biohazard bags 3. Alcohol based hand sanitizer and 1**% virkon**) 4. Knap sack sprayer and spray bottles 5. Detergent/soap 6. Jerry can of clean water 7. Ziploc bags 8. Permanent marker \| \| --- \| --- \|  1. **Precaution**   The environment and notably faeces, are potentially infectious materials. Exposures to biological samples may cause serious infection. All PPEs used on a farm should not be used on another farm as these can be a potential source of infection to the next farm. The farm owner or manager should be probed to ascertain any previous/current occurrence of infections. If there is evidence of any signs of illness affecting any of the units on a given poultry farm, then there should be no sample collection. All sample collectors should be trained on biosafety measures.   1. **Biosafety procedures and measures** 2. Plan carefully the number and location of sites to be visited per day. A maximum of 10 sites per day is recommended. 3. The team on each farm should comprise two trained personnel (experienced veterinarian and a laboratory technologists) who should be assisted by the farmer/caretaker. 4. Before setting off for the farm, sterile materials for sample collection should be placed in a disinfected plastic box with a lid. 5. Upon arrival at the sample collection site, establish rapport with the farmers/animal caretaker according to the acceptable cultural norms. Explain to him/her the purpose of your visit and what you are actually going to do. Explain why you need to exercise biosafety (protective) measures while collecting samples. 6. Each team member should wash hands using soap and water or rub hands with alcohol-based hand sanitizer. 7. Each team member must at all times while in the collection site put on appropriate PPE for the task. NB: only appropriately disinfected personnel and materials should be allowed on the farm. 8. Place the sample in a zip lock bag, and immediately keep in a cool box with ice packs. Place adequate adsorbent tissue on the bottom and on sides of the cool box. 9. Identify fresh chicken faecal dropping preferably the faeces dropped in your presence to sample and immediately put into the transport media after sampling. 10. When sample collection is done, labelled samples should be packaged appropriately in Ziploc bags which should be placed in a cooler box, accompanied by the data collection form. 11. Then, the team should package the reusable sample collection materials in a Ziploc bag and place them in the box for later sterilization. 12. Thereafter, the team should dispose the used pair of gloves and put on a new sterile set of gloves to use for disinfecting the box used to carry the sample collection materials. 13. When this is done, the team can disclose all the PPE, taking care to appropriately package the disposable PPE in a biohazard waste bag along with other waste generated during sample collection for subsequent disposal. Reusable PPEs should be adequately decontaminated with 1% virkon or 0.5% chlorine and packaged for further sterilization before being reused e.g. gumboots. 14. Collect all the waste materials (like used gloves, tissue paper, shoe covers) and put them in a biohazard bag for safe disposal when you return from the field. Never leave any waste materials in the site. 15. The team should then wash their hands thoroughly with soap and water or apply suitable alcohol hand sanitizer. It is advisable to carry some water in case there is no water in the site you are visiting. 16. Where applicable, park your vehicle outside the sample collection site. In case you enter site with a vehicle, disinfect the tyres with a Knap sack sprayer before leaving the farm. 17. Remember to thank the farmers/animal caretaker for allowing you to collect the samples and give him/her a tentative date you can give a feedback on the results of antimicrobial susceptibility tests of the samples collected. 18. If due to unforeseen circumstances, samples cannot be transported to the laboratory on the same day of collection, they should be stored at 4°C at the nearest facility. For example, temporary short-term storage in the field could be arranged with the district veterinary office. It is therefore important to verify when you are at the field if the district veterinary office has a refrigerator and space for short-term storing of your samples, in case need arises.   **Reference**  Draft National Biosafety and Biosecurity Bill  NEMA *policy on waste disposal*  WHO (2004). *Laboratory biosafety manual, World Health Organization.* Third edition.  CDC. *Biosafety in Microbiological and Biomedical Laboratories*. Centers for Disease Control and Prevention http://www.cdc.gov/biosafety/publications/bmbl5/index.htm.  WOAH Terrestrial Animal Health Code (2018b). Chapter 6.5 *Biosecurity procedures in poultry production.* |
| --- | --- | --- | --- | --- | --- | --- | --- | --- | --- | --- | --- | --- | --- | --- | --- | --- | --- | --- | --- | --- | --- | --- |

| SAMPLING OF FRESH FAECAL DROPPINGS FROM CHICKEN FARMSStandard Operating Procedure  \| SOP No. \| Document No /Version \| \| \| \| --- \| --- \| --- \| --- \| \| Title: \| Selection of chickens for sampling \| \| \| \| Replaces Document: \|  \| Date of Revision \|  \| \| Applied to: Veterinary Microbiology Laboratories \| Reviewed & approved by: \|  \|  \| \| Date of Original \|  \| Date for next review \|  \|  1. **Introduction**   Chickens at the end of their production cycle intended for food consumption should be selected for AMR surveillance in animals. This is because consumption of chicken products is generally high compared with other protein sources; chickens are an important source of foodborne infections globally; and antimicrobials are widely used in this sector, including some which are of concern to human health (1)   1. **Purpose**   The purpose of this SOP is to describe the procedures for selection of chicken unit for collection of fresh faecal samples from live healthy chicken at the end of their production cycle, as near as possible to the point at which they enter the food chain. The samples should be used for isolation of *Escherichia coli*. The isolates should be subjected to antimicrobial susceptibility testing (AST) against the selected antimicrobials panel in line with the Clinical and Laboratory Standards Institute (CLSI) recommendations.   1. **Responsibilities**   The fresh chicken faecal dropping for sampling should be selected randomly by experienced veterinarian. The veterinarian should ensure that selection is done on a farm which never had an outbreak within the last one week of production and with record of use of antibiotics on the farm. Farms with history of use of any dose(s) of antibiotics in the last seven days prior to the survey should not be sampled. Samples should be collected from farm unit with healthy chicken at the end of the production cycle at which they enter the food chain following the SOP.   1. **Materials and equipment**  \| 1. PPE kit (overalls, gum boots, aprons, powder free gloves of all sizes, disposable shoe covers) 2. Pens 3. Permanent markers 4. Data collection form 5. Cooler box(es) 6. Frozen ice packs 7. Paper towels 8. Biohazard bags \| 1. Disinfectant (**1%virkon** and 70% ethanol) 2. Knap sack sprayer and spray bottles 3. GPS reader with batteries 4. Detergent/soap 5. Jerry can of clean water 6. Ziplock bags 7. Falcon tubes 8. Sterile swab containers with transport media containing charcoal \| \| --- \| --- \|  1. **Precaution**   Selection should be done by an experienced veterinarian while in appropriate PPE. Only farm unit with healthy chicken about to enter food value chain should be selected randomly. Biosecurity practices on the farm should strictly be followed at all times.  The SOP should be used alongside the Biosafety and Biosecurity and sample collection SOPs.   1. **Procedures** 2. The selection process should involve veterinarian with PPE; 3. Select the unit/shed with the oldest flock at the end of production cycle about to enter food value chain; 4. Once inside the house/unit/shed, notionally divide the space in to ten equal quadrants; 5. Carefully observe the chicken from each quadrant for any abnormalities; 6. Select one chicken randomly for physical examination and ensure that a thorough systemic examination is done; 7. After a clinical examination, decide on the best sample to collect; 8. Identify freshly dropped faeces preferably the faeces dropped in your presence from the chickens in the quadrant. 9. Sample fresh faecal samples from the ten quadrants and pooled them to form one sample for the farm. From each farm, only one pooled sample should be considered.   **Reference**  1. Van Boeckel TP, Brower C, Gilbert M, Grenfell BT, Levin SA, Robinson TP, et al. Global trends in antimicrobial use in food animals. Proceedings of the National Academy of Sciences of the United States of America. 2015;112(18):5649-54. |
| --- | --- | --- | --- | --- | --- | --- | --- | --- | --- | --- | --- | --- | --- | --- | --- | --- | --- | --- | --- | --- | --- | --- |

| SAMPLE COLLECTION, LABELLING, PACKAGING TRANSPORTATION AND STORAGEStandard Operating Procedure  \| SOP No. \| Document No /Version \| \| \| \| --- \| --- \| --- \| --- \| \| Title: \| Sample collection, transportation and storage \| \| \| \| Replaces Document: \|  \| Date of Revision \|  \| \| Applied to: Veterinary Microbiology Laboratories \| Reviewed & approved by: \|  \|  \| \| Date of Original \|  \| Date for next review \|  \|  1. **Introduction**   Accurate and timely analysis is an important component of AMR surveillance and control. This necessitates that correct samples are selected, collected and submitted in a manner that maximizes the chances of isolating and identifying the target bacteria. This SOP provides information on the target chicken farm for sampling; and sample types, along with the guidelines on collection, handling, transportation as well as their storage.  The SOP should be used alongside the Uganda National Action Plan for AMR surveillance in animals and the World Organization for Animal Health (WOAH) Biosafety and Biosecurity manual.   1. **Purpose**   The purpose of this SOP is to describe the procedures for collection, handling, transportation and storage of faecal samples from poultry. These samples should be used for isolation of *Escherichia*. The isolates should be subjected to antimicrobial susceptibility testing against the commonly used antimicrobials in line with the Clinical and Laboratory Standards Institute (CLSI) recommendations.   1. **Responsibilities**   The sampling team shall comprise of experienced veterinarians and laboratory technologists. The team shall ensure that a correct sample is collected and packaged properly following the SOP and shall be transported to laboratory.   1. **Materials and equipment**  \| 1. Pens 2. Permanent markers 3. Note book 4. Data collection form 5. Cooler box(es) 6. Frozen ice packs 7. Paper towels 8. Biohazard bags 9. Disinfectant (**1%virkon** and 70% ethanol) 10. Knap sack sprayer and spray bottles 11. GPS reader with batteries \| 1. PPE kit (overalls, gum boots, aprons, powder free gloves of all sizes, disposable shoe covers) 2. Detergent/soap 3. Jerry can of clean water 4. Pair of scissors 5. Ziploc bags 6. Sterile spatula 7. Falcon tubes 8. Sterile swab containers with transport media containing charcoal \| \| --- \| --- \|  1. **Precaution**   Before collection, careful consideration should be taken to avoid contamination of the samples, exposing the collector to risks of zoonotic infections and spread of pathogens to the environment. The collector must wear appropriate protective gears (gloves, masks etc.), should collect specimens aseptically, and ensure proper packaging to avoid spillage. National and international waste management and disposal guidelines should be followed at all times   1. **Procedures** 2. ***Preparation for Sample collection:*** 3. The sampling process should involve two trained persons with PPE, working together as a team: 4. The first person (veterinarian) should administer the sample collection form (semi structured questionnaire) and decide on the right sample; 5. The second person (veterinarian or laboratory technologist) can collect the samples from the selected farm unit; 6. Fresh faecal samples should be collected using a sterile swab and put in Amies transport media containing charcoal. 7. Proper record keeping is critical at this stage as it should affect all sample identification and result interpretation. 8. Before starting the sampling, prepare the sampling team and define the duties of each person involved. 9. The sampling location should be preferably protected from wind, and all materials and equipment, such as tubes and boxes, must be placed on a dry and stable place 10. Arrange the sampling equipment for easy access. Make sure you have sufficient equipment for the number of samples you plan to collect. The sample collection tube should be labelled prior to sampling to avoid unnecessary delays. 11. ***Sample labelling*** 12. The semi structured questionnaire and sample container should have a similar code unique—matching code—to a particular farm sampled 13. Labelling of questionnaires and collection containers should have sequential poultry farm identification code (ID); followed by a sequential sample ID; followed by district name; followed by sample collection date (DD-MM-YYYY) i.e. PF001 (for poultry farm 1)/(District) e.g. WAK for Wakiso district /01-04-2023 (Date of sample collection). For example, PF001/WAK/01-04-2023. Note: P is poultry, F for Farm 14. ***Sample packaging and transportation*** 15. All samples should be secured in sample containers in a cool box with ice packs while all samples are being collected. 16. Double pack the tubes in sealable plastic bags to prevent leakage or cross contamination 17. Samples should be kept chilled (4 – 10^0^C), to prevent overgrowth of samples until they are delivered to the laboratory. 18. There should be no direct contact between ice and the sample. 19. The sample must be kept cold and transported to the laboratory within 72 hours of collection. Otherwise there are faecal bacteria associated with gas production that can rupture the sample tubes or displace the tube stoppers to cause leakage or cross contamination in the sealable bag.   **Reference**  WOAH, 2013: *Manual of Diagnostic Tests and Vaccines for Terrestrial Animals*  Web site: www.fao.org/emergencies/programmes/CMC-AH |
| --- | --- | --- | --- | --- | --- | --- | --- | --- | --- | --- | --- | --- | --- | --- | --- | --- | --- | --- | --- | --- | --- | --- |
