# Supplementary material for: Prevalence and factors associated with multidrug resistant Escherichia coli carriage on chicken farms in west Nile region in Uganda: A cross-sectional survey
Source: PLOS Glob Public Health. 2025 Jan 16;5(1):e0003802. doi: 10.1371/journal.pgph.0003802 (PMC11737776; doi:10.1371/journal.pgph.0003802)
Supplement: Attachment — Submitted filename: Peer review comments from Mateus_MDR.docx [file pgph.0003802.s005.docx]

**Peer review comments from Mateus**

**Title:**

**Prevalence and factors associated with Multidrug Resistant *Escherichia coli* carriage on chicken farms in west Nile region in Uganda: A cross-sectional survey.**

**Abstract**

Line 18-19: “A total of 158 *E. coli* strains were isolated from 158 chicken farms”. What is the study population (chicken farms or E. coli strains)? 158 strains for 158 chicken farms? Does it mean each chicken with its strain?

**Introduction**

Line 41-42: AMR reported in animals or in humans? As the authors have studied AMR in poultry, it is important to specify if this sentence is related to human or animals or both.

Line 48: predisposition of emergence AMR to humans or animals?

Lines 52-54: Is there any existing policy for AMR control in Uganda? If yes, better to start this before speaking about laxity of policy implementation.

Line 59: Fix the grammar issue in this sentence.

Line 61: There is repetition of “the”

Line 55-62: “Gender determines the division of labour in many contexts especially in Low- and Middle-Income Countries (LMICs). In most of the farming communities in Sub Saharan Africa (SSA), female gender particularly, women take care of sick animals not destined for slaughter while the male gender owns the farms. Compared to the male gender counterparts, females gender also plays critical roles in household activities and taking care of children including household water, sanitation and hygiene (WASH) activities. These roles increases their exposure to and spread of resistant zoonotic pathogens including the drug resistant ones“ => is this paragraph relevant to this study? Are the authors studying the level of AMR between males and females?

Line 66: recent past? => it is better to be precise about a period (recent past years, recent past months, ect.).

Line 70: The sentence “… distributed among humans, animals and environment” should read “… distributed among humans, animals, and environment”.

**Methods**

Line 111-117: What is the study population here (poultry or humans (farmers))? What is the purpose of the questionnaire for this study? It is important to be clear about that. How the households were selected to be included in the study?

Lines 118-131: Sampling: this is good details about selection of the chickens. So, what about the farmers who attended the survey questionnaire?

Lines 157-159: Are these demographic characteristics for chickens or for farmers?

Lines 183-184: The following sentence looks like a result and should be in the results section: “All the farms sampled tested positive for *E.* *coli* and an isolate was chosen from each of the 158 farms for AST”.

**Results**

Table 2: merge some rows for the column of variables to match the sub-categories.

Table 3: merge some rows for the column of variables to match the sub-categories.

Fig 1: It will be good to add labels to your figure. As it is, it looks like all the 3 colors are equally distributed.

Line 292-293: The sentence “being higher on female managed farms than male managed farms (68.7% vs 31.3% respectively)” should read “being higher for females than males managing farms (68.7% vs 31.3% respectively)”.

**Discussions**

Lines 321-329: This paragraph is a repetition of the results. The authors can think if it necessary to keep it here. Otherwise, I would suggest to remove it as it is already stated in the results. And in the results, think about rephrasing the sentence about gender. As it is written, it looks like the MDR was studied for people rather than for chickens.

Line 356: The sentence: “The low prevalence of MDR *E. coli* on male managed farms …” should read “The low prevalence of MDR *E. coli* for farms managed by males …”

Line 370: Please think about rephrasing this sentence: “… prevalence of MDR *E. coli* among female gender …”. What you mean by female gender here? (human or animal?). You are studying MDR in chicken, not in human. Go through the whole text and be precise about this.

Lines 375-376: “the observed high prevalence of MDR *E. coli* especially on farms managed by females could impact negatively on maternal and child nutrition”. => this needs further details. It is still not clear where it comes from.

Line 387: The sentence “… in Makerere University…” should read “… at Makerere University …”

Lines 400-419: This paragraph is a repetition of what you stated above. Think about removing it or shorten it to essential information.

Line 445: Be sure you have stated clearly in the methods that the analysis was phenotypic rather than genotypic.

Add in the study limitation that the study did not look at AMR transmission from chicken to human and that this can be explored in future researches.

**Conclusion**

Line 455: “… protected against *E. coli* resistance …” => this is an over claim. May be say “… reduce E. coli resistance …”.

**General comments:**

This is a very good study that add value to the literature. Authors can consider the above comments to improve the paper.
