# Supplementary material for: Prevalence and factors associated with multidrug resistant Escherichia coli carriage on chicken farms in west Nile region in Uganda: A cross-sectional survey
Source: PLOS Glob Public Health. 2025 Jan 16;5(1):e0003802. doi: 10.1371/journal.pgph.0003802 (PMC11737776; doi:10.1371/journal.pgph.0003802)
Supplement: Attachment — Submitted filename: Response to Reviewers.docx [file pgph.0003802.s006.docx]

**Rebuttal Letter**

Dear Ben Pascoe
(Academic Editor, PLOS Global Public Health),

We appreciate the insightful comments and suggestions provided by the reviewers. We confirmed that our submission contains all raw data required to replicate the results of the study.

Below, we provide a point by point response to each of the concerns raised and made the necessary revisions to the manuscript.

**Abstract:**

**Reviewer Comment:**

*Line 18-19: “A total of 158 E. coli strains were isolated from 158 chicken farms”. What is the study population (chicken farms or E. coli strains)? 158 strains for 158 chicken farms? Does it mean each chicken with its strain?*

**Response:** We appreciate the need for clarity here. We modified the sentence to reflect that *E. coli* was isolated from each of the 158 chicken farms sampled. The sample size was 158 chicken farms and *E. coli* was isolated from each of the pooled sample from each farm. Please see lines 18 and 19 on page 1 of the revised manuscript.

***Introduction***

**Reviewer Comment:** Line 41-42: AMR reported in animals or in humans? As the authors have studied AMR in poultry, it is important to specify if this sentence is related to human or animals or both.

**Response:** Thank you for this observation. We have revised the sentence to clarify that the AMR being referred to pertains specifically to the prevalence of AMR in animals, particularly poultry. However, AMR prevalence in humans have been cited in the manuscript to indicate the potential spillover of resistant strains from animals to humans as reported by other studies. We have ensured that the distinction between AMR in animals and humans is clearly communicated throughout the manuscript. Please see lines 43 and 44 on page 2 in the revised manuscript.

**Reviewer Comment:** Line 48: predisposition of emergence AMR to humans or animals?

**Response:** We appreciate the need for clarity here. The sentence has been modified to specify that the predisposition refers to the emergence of AMR in animals, particularly in the poultry industry. The revised text now more explicitly delineates the context of AMR emergence. Please see line 48 on page 2 of the revised manuscript.

**Reviewer Comment:** Lines 52-54: Is there any existing policy for AMR control in Uganda? If yes, better to start this before speaking about laxity of policy implementation.

**Response:** We have revised this section to acknowledge the existing policies on AMR control in Uganda. The revised text now begins with a brief mention of the relevant policy (AMR National Action Plan (NAP) in 2018) before discussing the challenges and laxity in the implementation. This adjustment provides a more balanced perspective on the issue with relevant citation. Please see lines 53 – 55 on page 2 of the revised manuscript.

**Reviewer Comment:** Line 59: Fix the grammar issue in this sentence.

**Response:** We have corrected the grammatical error in line 59. The sentence has been revised for clarity and correctness. It now reads as ‘In most farming communities in Sub-Saharan Africa (SSA), women, in particular, are responsible for caring for sick animals that are not intended for slaughter, while men majorly own the farms’ Please see lines 59 - 61 on page 3 of the revised manuscript.

**Reviewer Comment:** *Line 61: There is repetition of “the”*

**Response:** We have corrected this repetition in line 64 on page 3 by removing “the”.

**Reviewer Comment:** *Line 55-62: “Gender determines the division of labour in many contexts especially in Low- and Middle-Income Countries (LMICs). In most of the farming communities in Sub Saharan Africa (SSA), female gender particularly, women take care of sick animals not destined for slaughter while the male gender owns the farms. Compared to the male gender counterparts, females’ gender also plays critical roles in household activities and taking care of children including household water, sanitation and hygiene (WASH) activities. These roles increase their exposure to and spread of resistant zoonotic pathogens including the drug resistant ones “=> is this paragraph relevant to this study? Are the authors studying the level of AMR between males and females?*

**Response:** We have revised the sentence of lines 55 – 62. We acknowledge that the study primarily focuses on animals (poultry) rather than humans. However, it is important to recognize the role of human behaviour in the transmission and spread of AMR on farms. The paragraph in question (Lines 55-62) highlights the gender-specific roles within farming communities, particularly in Low- and Middle-Income Countries (LMICs), such as those in Sub-Saharan Africa (SSA). These roles—where women are often responsible for caring for sick animals and managing household activities, including water, sanitation, and hygiene (WASH)—are significant as they may increase the risk of exposure to and transmission of resistant zoonotic pathogens. While this study does not specifically compare AMR levels between males and females, it is essential to understand these gender-based dynamics as they contribute to the broader context of AMR spread on farms. *“Our study found a significant difference in prevalence of MDR E. coli between female and male manged farms (68.7% vs 31.3%). At multivariable analysis after adjusting for factors included in the model, chicken farms managed by males had a lower prevalence of MDR E. coli (APR = 0.72, 95% CI: 0.55 – 0.93) compared to those managed by females”.* Please see lines 58 – 65 on page 3 of the revised manuscript.

**Reviewer Comment:** *Line 66: recent past? => it is better to be precise about a period (recent past years, recent past months, ect.).*

**Response:** We have corrected line 66 to read “However, strains known to cause illnesses in animals and humans have emerged in the recent past years”. Please see line 70 on page 3 of the revised manuscript.

**Reviewer Comment:** *Line 70: The sentence “… distributed among humans, animals and environment” should read “… distributed among humans, animals, and environment”.*

**Response:** This sentence has been corrected to reflect the suggested statement: … distributed among humans, animals, and environment. Please see line 73 on page 3 of the revised manuscript.

***Methods***

**Reviewer Comment:** *Line 111-117: What is the study population here (poultry or humans (farmers))? What is the purpose of the questionnaire for this study? It is important to be clear about that. How the households were selected to be included in the study?*

**Response:** Lines 111–117 have been revised to clarify the study population, specifying that the primary focus was on chicken farms. The secondary units included chickens, from which fresh faecal samples were collected, and humans (farm owners/managers), who were surveyed using semi-structured questionnaires to determine risk factors associated with MDR *E. coli*. Please see lines 116 – 120 on page 5 of the revised manuscript.

**Reviewer Comment:** *Lines 118-131: Sampling: this is good details about selection of the chickens. So, what about the farmers who attended the survey questionnaire?*

**Response:** In regards to farmer selection for the survey in lines 118 – 131, on each farm a household member who met the study's selection criteria was purposively chosen for the interview. Please see lines 123 - 124 on page 5 of the revised manuscript.

**Reviewer Comment:** *Lines 157-159: Are these demographic characteristics for chickens or for farmers?*

**Response:** The sentence in lines 157–159 has been revised to clearly distinguish the demographic characteristics of both the farmers and the chickens. Please see lines 167 – 169 on page 7 of the revised manuscript.

**Reviewer Comment:** *Lines 183-184: The following sentence looks like a result and should be in the results section: “All the farms sampled tested positive for E. coli and an isolate was chosen from each of the 158 farms for AST”.*

**Response:** The sentence in lines 183 – 184, we revised it to exclude *“All the farms sampled tested positive for E. coli”*. Please see lines 193 and 194 on page 8 of the revised manuscript.

***Results***

**Reviewer Comment:**

*Table 2: merge some rows for the column of variables to match the sub-categories.*

*Table 3: merge some rows for the column of variables to match the sub-categories.*

**Response:** The rows of Table 2 and 3 have been merged as needed to align with the sub-categories in the tables.

**Reviewer Comment:** *Fig 1: It will be good to add labels to your figure. As it is, it looks like all the 3 colors are equally distributed.*

**Response:** Fig 1 has been labelled and color codes have been partitioned.

**Reviewer Comment:** *Line 292-293: The sentence “being higher on female managed farms than male managed farms (68.7% vs 31.3% respectively)” should read “**being higher for females than males managing farms (68.7% vs 31.3% respectively)”.*

**Response:** We are grateful for the suggestion, we have corrected the sentence to read *“being higher for females than males managing farms (68.7% vs 31.3% respectively)”*. Please see lines 307 and 308 on page 14 of the revised manuscript.

***Discussions***

**Reviewer Comment:** *Lines 321-329: This paragraph is a repetition of the results. The authors can think if it necessary to keep it here. Otherwise, I would suggest to remove it as it is already stated in the results. And in the results, think about rephrasing the sentence about gender. As it is written, it looks like the MDR was studied for people rather than for chickens.*

**Response:** We appreciate your feedback on Lines 321-329. We believe that this paragraph is important for providing essential background information that supports the discussion. However, we have revised the statement on gender to ensure clarity and to avoid any misunderstanding that the study focused on MDR in humans rather than chickens. Farmers were interviewed to determine the risk factors of MDR on chicken farms. Please see lines 346 and 348 on page 17 of the revised manuscript.

**Reviewer Comment:** *Line 356: The sentence: “The low prevalence of MDR E. coli on male managed farms …” should read “The low prevalence of MDR E. coli for farms managed by males …”*

**Response:** We have corrected this sentence to read “The low prevalence of MDR *E. coli* for farms managed by males...” Please see lines 378 on page 380 of the revised manuscript.

**Reviewer Comment:** *Line 370: Please think about rephrasing this sentence: “… prevalence of MDR E. coli among female gender …”. What you mean by female gender here? (human or animal?). You are studying MDR in chicken, not in human. Go through the whole text and be precise about this.*

**Response:** We have rephrased the sentence in line 370 to “… prevalence of MDR *E. coli* on female managed farms …”. We referred to the gender of the farmer. MDR reported in this study was from chicken farms however, we interviewed farm owners/managers to determine MDR *E. coli* risks on the farms included in the study. Furthermore, the study points out the role of human behaviour in the transmission and spread of AMR on farms.

**Reviewer Comment:** *Lines 375-376: “the observed high prevalence of MDR E. coli especially on farms managed by females could impact negatively on maternal and child nutrition”. => this needs further details. It is still not clear where it comes from.*

**Response:** We have revised the sentence for clarity and have expanded the explanation as follows:

“Therefore, the observed high prevalence of MDR *E. coli*, particularly on farms managed by females, raises concerns about the potential negative effects on maternal and child nutrition, as it could lead to increased nutrition insecurity, compromised health outcomes, and hindered economic growth”. Please see lines 400 – 403 on page 19 of the revised manuscript.

**Reviewer Comment:** *Line 387: The sentence “… in Makerere University…” should read “… at Makerere University …”*

**Response:** We have corrected the sentence in line 387 to read “….at Makerere University….” as suggested. Please see lines 414 on page 19 of the revised manuscript.

**Reviewer Comment:** *Lines 400-419: This paragraph is a repetition of what you stated above. Think about removing it or shorten it to essential information.*

**Response:** We have addressed your concern about the repetition in Lines 400 – 419 by significantly shortening the paragraph. The revised version retains the essential information, eliminating redundant details while ensuring that the key points are clearly conveyed. We focused on maintaining the important connections between gender roles, the management of farms by females, and the associated public health implications. We hope this revision meets your expectations. Please see lines 430 and 442 on page 20 of the revised manuscript.

**Reviewer Comment:** *Line 445: Be sure you have stated clearly in the methods that the analysis was phenotypic rather than genotypic.*

*Add in the study limitation that* *the study did not look at AMR transmission from chicken to human and that this can be explored in future researches.*

**Response:** Line 445. We have clarified in the methods that the study focused on phenotypic analysis (206). We stated in the study limitation that the study did not consider AMR transmission from chicken to humans. We suggested genotypic and molecular studies should be considered not only understanding transmission from chicken to humans but also the flow of resistomes across the human, animal, and environment interface. Please see lines 465 - 466 on page 21 and lines 483 – 485 on 22 of the revised manuscript.

***Conclusion***

**Reviewer Comment:** *Line 455: “… protected against E. coli resistance …” => this is an over claim. May be say “… reduce E. coli resistance …”.*

**Response:** We have rephrased the sentence in line 455 to “…...posed reduced risk of *E. coli* resistance….” as suggested. Please see lines 479 on page 22 of the revised manuscript.

**Reviewer 2:**

**Reviewer Comment:** *A recommendation would be to provide details on the stool sample collection protocols eg who collected (qualified or not), standard operating protocols eg what measures were in place to ensure reproducibility of the sample collection process.*

**Response:** We have included additional details regarding the fecal sample collection protocols in the revised manuscript. Specifically, we have clarified that all samples were collected by qualified animal health officers following standardized operating procedures. These procedures included measures to ensure the reproducibility of the sample collection process, such as consistent training for collectors, the use of sterile equipment, and adherence to strict handling and transport protocols to maintain sample integrity. Please see lines 138 - 140 on page 6 of the revised manuscript.

We also provided Standard Operating Procedure considered during faecal sample collection as support document. Please see lines 494 on page 22 of the revised manuscript.

We hope that these revisions address the concerns raised by the reviewers. We believe that the changes have strengthened the manuscript, and we are grateful for the opportunity to improve our work.

Thank you for considering our revised manuscript for publication.

Sincerely,

Dr Ceaser Adibaku Nyolimati
Baylor Children’s Foundation Uganda
+256 774 047958,

[cnyolimati@gmail.com](mailto:cnyolimati@gmail.com)
