# Supplementary material for: Prevalence and factors associated with multidrug resistant Escherichia coli carriage on chicken farms in west Nile region in Uganda: A cross-sectional survey
Source: PLOS Glob Public Health. 2025 Jan 16;5(1):e0003802. doi: 10.1371/journal.pgph.0003802 (PMC11737776; doi:10.1371/journal.pgph.0003802)
Supplement: Attachment — Submitted filename: Peer review comments from Mateus_MDR-2.docx [file pgph.0003802.s007.docx]

**Peer review comments from Mateus**

**Title:**

**Prevalence and factors associated with Multidrug Resistant *Escherichia coli* carriage on chicken farms in west Nile region in Uganda: A cross-sectional survey.**

**Abstract**

OK

**Introduction**

OK

**Methods**

Line 123-124: This sentence “On each farm, a household member who met the study's selection criteria was purposively chosen for 124 the interview” is repeated in the following sub heading (line 140-141).

- State clearly in the methods that the analysis was phenotypic rather than genotypic.

**Results**

Line 292-294: Fig 1: Thanks for making it clear. Now it looks like a table. It should be Table 4 instead.

**Discussions**

OK

**Conclusion**

OK

**General comments:**

This is a very good study that add value to the literature. I am okay for it to be published after fixing these very minor comments.
