# Supplementary material for: Prevalence and factors associated with multidrug resistant Escherichia coli carriage on chicken farms in west Nile region in Uganda: A cross-sectional survey
Source: PLOS Glob Public Health. 2025 Jan 16;5(1):e0003802. doi: 10.1371/journal.pgph.0003802 (PMC11737776; doi:10.1371/journal.pgph.0003802)
Supplement: Attachment — Submitted filename: Response to Reviewers.docx [file pgph.0003802.s008.docx]

Dear Editorial Manager,

PLOS Global Public Health,

We appreciate the reviewers' thorough feedback and constructive comments. Below are our responses to the requested revisions:

**Methods:**

**Reviewer Comment:** *Clarify that the analysis conducted was phenotypic rather than genotypic.*

**Response:** The text in the Methods section has been updated to specify that the analysis conducted was phenotypic by revising the sub heading ‘‘***Phenotypic Antimicrobial Susceptibility Testing’’*** and the subsequent sentence. The revised sentence now reads: ‘‘*Phenotypic antimicrobial susceptibility testing (AST) for E. coli isolates was conducted according to the standard operating procedure, using Kirby-Bauer disc diffusion method on Mueller-Hinton agar method and interpreted as either resistant, intermediate or susceptible according to clinical breakpoints as defined by the Clinical and Laboratory Standards Institute (CLSI) 2023 guidelines (version 33)’'*.

**Reviewer Comment:** *Methods (lines 123–124 and 140–141): Remove the repetition of the sentence describing the selection criteria for interviewees.*

**Response:** The repetitive sentences in the lines 123 - 124 under study design have been removed. The selection criteria for interviewees are now described only once in lines 139 – 140 in the sampling procedure section to enhance conciseness and avoid redundancy.

**Result:**

**Reviewer Comment:** *Adjust Figure 1 and present it as Table 4 for consistency.*

**Response:** *Figure 1 has been converted into a tabular format and is now presented as Table 4 in the Results section. The accompanying text and table caption have been adjusted to ensure clarity and alignment with the data.*

We hope these revisions meet the reviewers' expectations and look forward to proceeding with the next steps in the publication process.

Best regards,

Dr Ceaser Adibaku Nyolimati
Baylor Children’s Foundation Uganda
+256 774 047958,

[cnyolimati@gmail.com](mailto:cnyolimati@gmail.com)
